# BRASH Syndrome: A Case Report

**DOI:** 10.7759/cureus.97221

**Published:** 2025-11-19

**Authors:** Punita K Basra, Tarek Hammad, Adnan Adnan, Tirthkumar A Patel

**Affiliations:** 1 Medicine and Surgery, Northampton General Hospital, Northampton, GBR; 2 Cardiology, Northampton General Hospital, Northampton, GBR; 3 Acute Medicine, Northampton General Hospital, Northampton, GBR; 4 General Internal Medicine, University Hospitals of Leicester National Health Service (NHS) Trust, Leicester, GBR

**Keywords:** av nodal blockade, bradycardia, brash syndrome, hyperkalemia, renal failure

## Abstract

BRASH syndrome, characterized by Bradycardia, Renal Failure, AV (atrioventricular) nodal blockade, Shock, and Hyperkalemia, is a newly recognized clinical entity arising from the synergy between AV nodal blockade and hyperkalemia. This case reported an elderly female patient presented with dizziness and confusion and a complex medical history, including chronic kidney disease, atrial fibrillation, chronic obstructive pulmonary disease (COPD), and heart failure, with a medication regimen including Digoxin and Bisoprolol. Clinical examination revealed severe bradycardia and hypotension, necessitating high-flow oxygen. Diagnostic tests confirmed bradycardia, kidney and liver dysfunction, hyperkalemia, and acidosis, strongly suggesting BRASH syndrome, while other differentials were considered and ruled out. Treatment involved a temporary pacemaker, anti-hyperkalemic measures, and careful monitoring, leading to improved heart rate and resolution of symptoms. Further research is needed to establish consistent diagnostic criteria, but understanding the pathophysiology as hypoperfusion stemming from hyperkalemic potentiation of AV blocker activity can aid in quicker recognition and improve patient outcomes.

## Introduction

The symptoms of this illness, which include bradycardia, renal failure, atrioventricular nodal blockage, shock, and hyperkalemia, are combined to form the acronym BRASH (Bradycardia, Renal failure, Atrioventricular nodal blockade, Shock, and Hyperkalemia). This acronym refers to a state that develops when negative chronotropic and/or inotropic drugs are combined with renal insufficiency. The pathogenesis of BRASH syndrome stems from the mutually reinforcing effects of hyperkalemia and atrioventricular nodal blockade. The glomerular filtration rate is lowered and renal perfusion is slightly decreased due to a benign occurrence like moderate dehydration. This insult causes the accumulation of renal-cleared cardiovascular medicines, including antiarrhythmics, non-dihydropyridine calcium channel blockers, and beta-blockers [1].

Beta-blockers and calcium channel blockers have been around since the 1960s and are commonly used to treat illnesses like atrial fibrillation, hypertension, and coronary artery disease. The atrioventricular node is the site of action for both medicines, lowering the heart rate. Similar to several medications, atrioventricular nodal blockers are linked to unfavourable effects. The most typical ones include dyspnea, tiredness, lightheadedness, and disturbed sleep. But if left undiagnosed and untreated, BRASH syndrome is an underappreciated consequence that can cause severe morbidity, including multi-system organ failure and cardiovascular collapse [2].

Since this is a recently defined illness, not much is known about the epidemiology of this syndrome; case reports do suggest that older people with underlying cardiac and renal failure are more likely to have this syndrome. Multiple atrioventricular nodal blockers considerably raise the patient's likelihood of having this condition. Acute renal damage as well as hyperkalemia are two additional risks associated with angiotensin-converting enzyme inhibitors and angiotensin receptor blockers [3].

Here, we describe the case of a patient who presented with hypotensive urgency and congestive heart failure and went on to develop BRASH syndrome.

## Case presentation

**Figure 1:**
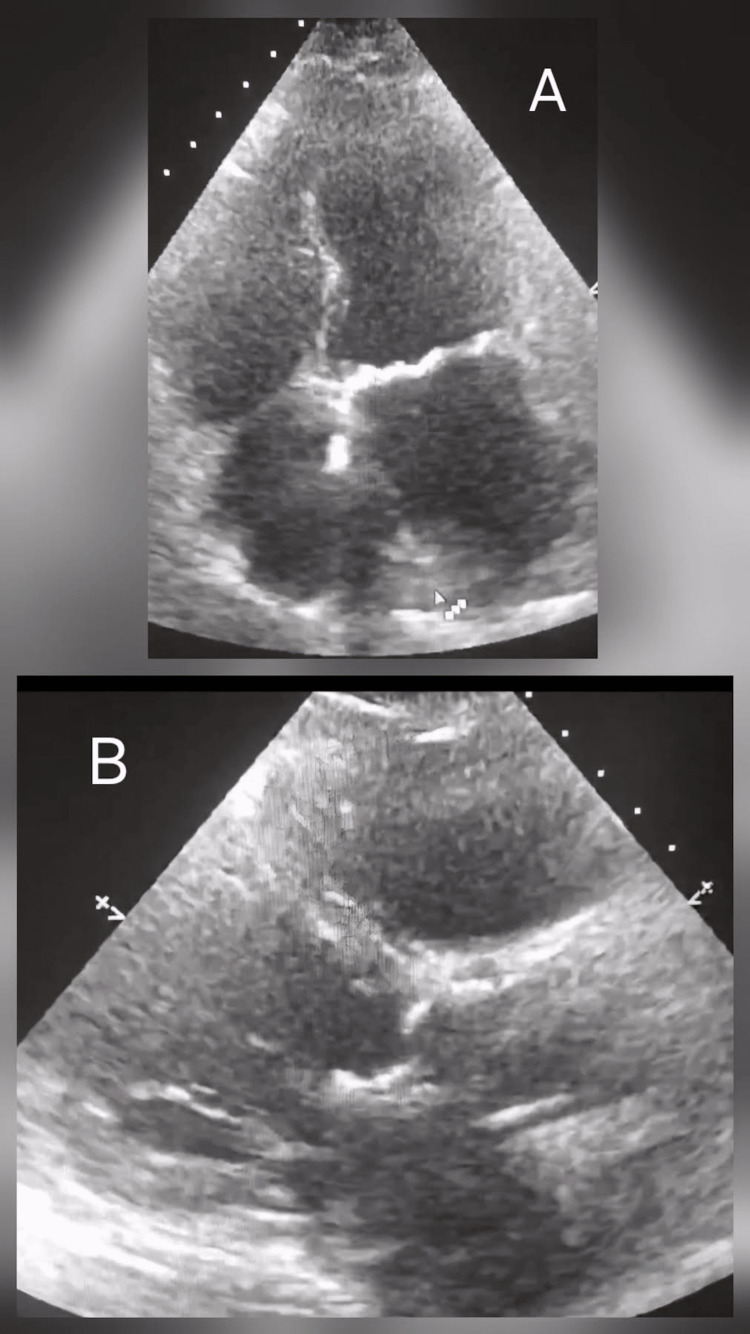
ECHO image (A) apical four chamber showing left ventricle (LV) is normal in size and function. Reduced right ventricle (RV), dilated atria. (B) Parasternal long axis view showing LV is normal in size and function with dilated atria

A female patient in her 80s presented to us with complaints of dizziness and confusion. She had a known history of chronic kidney disease with a glomerular filtration rate of 29 mL/min/1.73m². She was also diagnosed with atrial fibrillation and was on Digoxin, Apixaban, and Bisoprolol for this condition. Additionally, she had chronic obstructive pulmonary disease and hypertension, for which she was taking oral inhalers and Candesartan, respectively. Furthermore, she was being treated for heart failure with Bumetanide and had a mood disorder for which she was prescribed Mirtazapine. An ECHO (echocardiogram) on admission showed a non-dilated left ventricle with good function, severe mitral and tricuspid regurgitation and moderate aortic regurgitation (Figure 1).

Upon examination, the patient was confused and disoriented, not knowing who or where she was. Her chest was clear on auscultation with no signs of any heart murmur. Her observations on admission are shown in Table 1.

**Table 1:** Laboratory findings and reference range

	Laboratory values	Normal Range Adult female
Potassium	6.8 mEq/L	3.5-5.2 mEq/L
Urea	39 mg/dL	6-24 mg/dL
Creatinine	353 µmol/L	52.2 to 91.9 µmol/L
Glomerular filtration rate	10 mL/min/1.73m²	>90 mL/min/1.73m²
Alanine transaminase	509 U/L	10-60 U/L
Troponin I	60 ng/mL	0-0.04 ng/mL
Serum Lactate	8 mmol/L	0.5-2.2 mmol/L
Digoxin	1.4 µg/L	0.8-2.0 µg/L

Investigations

**Figure 2:**
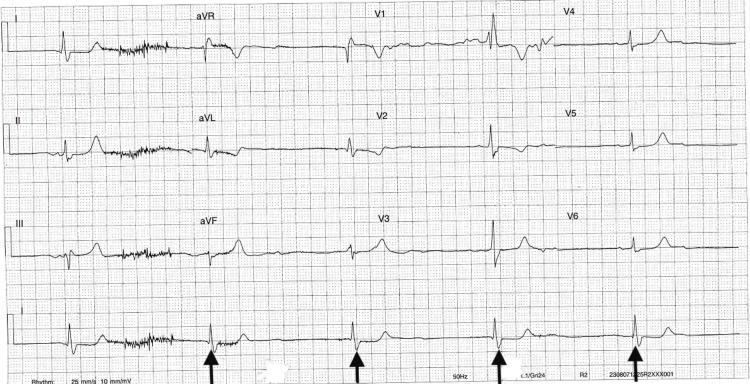
ECG of patient showing a 2:1 block

An electrocardiogram confirmed bradycardia with a complete heart block (Figure 2). Laboratory tests (as shown in Table 2) revealed abnormal kidney and liver function, with elevated troponin and lactate levels. A chest X-ray showed hazy upper zone congestions in keeping with pulmonary congestive/ venous diversion and multiple rib fractures.

**Table 2:** Observations on admission LPM: liters per minute; E: eyes; V: verbal response; M: motor.

Observations	
Blood pressure	84/64 mmHg
Saturation	100% on 10 LPM via nasal cannula
Respiration rate	18 bpm
Temperature	36.1°C
Heart rate	30 bpm
Glasgow Coma Score	14/15 (E 4 , V 4 , M 6)

Differential diagnosis

It can be difficult to differentiate BRASH syndrome from other conditions that may mimic its presentation. There can be multiple differentials, including AV blocker toxicity, cardiogenic shock, hypothermia and isolated hyperkalemia. In our case, the patient's digoxin levels were normal on admission (as demonstrated in Table 1) and there was no history of drug overdose, which helped us rule out drug toxicity as a differential. Furthermore, although our patient's blood test results indicated hyperkalemia, this was not isolated and was accompanied by digoxin use and symptoms of shock. Hypothermia may also present with bradycardia and shock, but there was no cold exposure in the presentation history and on admission, the patient's temperature was acceptable (as shown in Table 2). Finally, although the symptoms of shock, bradycardia and renal failure are also present in cardiogenic shock, it would not explain the hyperkalemia. Our patient presented with a combination of profound bradycardia, renal failure, shock and hyperkalemia and in the context of her medication history involving Digoxin and Bisoprolol, it strongly indicated BRASH syndrome as the leading diagnosis.

Treatment

**Figure 3:**
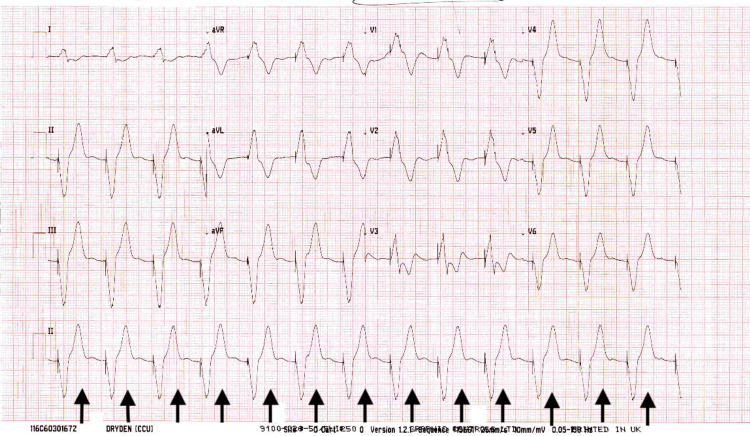
ECG showing a paced rhythm after temporary pacing wire insertion.

A temporary pacemaker was successfully inserted to address the severe bradycardia (Figure 3). Additionally, medical management involved holding Digoxin and beta-blockers, fluid resuscitation and two doses of atropine were administered. Anti-hyperkalemic measures involving insulin, glucose and calcium gluconate were initiated, and urine output was carefully monitored. These measures effectively managed the patient's hyperkalemia. As a result, the patient's clinical condition improved over the next few days and her confusion resolved. The treatment successfully addressed the underlying issues, resulting in significant clinical improvement of the patient's condition.

## Discussion

This syndrome is a unique condition that is increasingly common as a cause of profound bradycardia because of the comorbidities associated with age and the availability of new medications that target cardiac output. A vicious cycle of severe bradycardia and hyperkalemia is brought on by the interaction of atrioventricular nodal blockage and renal failure. There is a theory that, despite being widespread, it is frequently overlooked and leads to incorrect diagnoses in intensive care units [4]. This case report describes the presentation, diagnosis and successful treatment of BRASH syndrome in an elderly female patient with profound bradycardia, hyperkalemia and shock.

BRASH syndrome can be brought on by a number of factors, including dehydration, acute kidney injury and antihypertensives. Hypovolemia, however, is the most frequent trigger for BRASH syndrome [5]. One of the numerous potential causes of decreased perfusion that can result in an acute kidney injury (serum creatinine by ≥0.3 mg/dL from baseline) is hypovolemia (whether from decreased intake or increased loss). Acute kidney injury then causes less potassium to be excreted, which makes the body hyperkalemic (K+ level ≥5.5 mg/dL). It is believed that the hyperkalemic condition will collectively intensify the body's response to atrioventricular blocking medications, aggravating bradycardia (heart rate) [6]. Bradycardia can also be brought on by atrioventricular nodal blocking medications and hyperkalemia. Bradycardia alone, however, can cause acute kidney injury, decreased cardiac output and decreased renal perfusion in cases of underlying renal dysfunction. These factors worsen hyperkalemia and set off a vicious cycle of multiorgan dysfunction known as BRASH syndrome [7].

Therefore, the presented case underscores the critical role of rapid and accurate diagnosis in managing BRASH syndrome. The complex interplay of renal impairment, bradycardia and medication accumulation necessitates a nuanced approach. Healthcare professionals must remain vigilant, especially when dealing with elderly patients with multiple comorbidities and a history of medications affecting cardiac function. Early recognition, prompt discontinuation of atrioventricular nodal-blocking agents, and targeted interventions for hyperkalemia are pivotal in successfully managing BRASH syndrome and mitigating its potentially life-threatening consequences.

Once other potential causes have been ruled out, the diagnosis of BRASH syndrome is primarily based on the clinical manifestations, electrocardiogram findings and a comprehensive metabolic panel. When dealing with patients who have a history of atrioventricular nodal blocking medication, elevated serum potassium, refractory bradycardia or renal failure, the level of suspicion should be high [3]. In our case, the absence of other significant diagnoses, such as sepsis, and the existence of the previously listed set of symptoms led to a clinical suspicion of BRASH syndrome. Our patient was taking beta-blockers continuously and had chronic renal failure, both of which increase the risk of developing BRASH syndrome. Furthermore, hyperkalemia is a significant factor in the development of bradyarrhythmia. But contrary to what has been documented in the literature, our patient's electrocardiogram revealed severe bradycardia without any of the hallmarks of hyperkalemia, such as peaked T waves or sine waves [8].

As described in the *Journal of Emergency Medicine*, the most common mistake in managing BRASH syndrome is fixating on a singular component of the syndrome while leaving other aspects overlooked, resulting in patient harm. Understanding the pathophysiology of BRASH syndrome can aid us in coordinating a treatment plan that addresses every aspect of the syndrome. Thus, the treatment involves simultaneously addressing several problems [3].

Stopping all atrioventricular nodal-blocking medications is the first step in treating BRASH syndrome. Taking care of hyperkalemia is crucial. Intravenous calcium can be used to treat hyperkalemia quickly by stabilising the heart membrane and increasing cardiac output, but it does not lower the concentration of extracellular potassium. Combination treatments that enhance total body clearance or encourage the intracellular shift of potassium enhance response even more. If i.v. (intravenous) calcium fails to resolve the bradycardia, we can consider treatment with epinephrine infusion. This will have a two-fold effect. Firstly, it will increase the heart rate and cardiac output, thereby improving hemodynamics.

A point of note is that the standard cardiac life support algorithm for bradycardia will fail to treat hyperkalemia aspect of BRASH syndrome as it does not include calcium. In BRASH syndrome, we need to target both bradycardia and hyperkalemia with a combination of atropine/epinephrine/pacemaker and calcium. Therefore, patients do not get a chance to respond to medical treatment, resulting in unnecessary pacemaker placement, solidifying the importance of recognising BRASH syndrome [9].

Patients may also present with severe bradycardia but can be normotensive. In such cases, patients are compensating for bradycardia with pronounced vasoconstriction. These patients still suffer from malperfusion and so treating bradycardia remains important in re-establishing perfusion in renal function. We must also be vigilant with patients' fluid status, as they can be both hypovalemic or hypervolemic. 

Finally, we must consider a definitive treatment for hyperkalemia. Such treatments include potassium-wasting diuretics or short-term dialysis. Consideration may be given to a transcutaneous pacemaker if the patient's bradycardia persists [10] as seen in our patient.

## Conclusions

In conclusion, BRASH syndrome is a clinical diagnosis that is not well understood, and its symptoms are frequently mistaken for straightforward hyperkalemia. This confusion is understandable though, because there is a wide range in the presentation of BRASH syndrome, and more research is required to establish reliable diagnostic criteria. This instance highlights an important lesson, highlighting the significance of quick, customised interventions as well as a thorough understanding of this condition. By staying informed and vigilant, healthcare providers can significantly enhance the outcomes for patients afflicted with BRASH syndrome, ensuring a higher quality of care and improved overall patient well-being.

Regarding the duration or necessity of restarting atrioventricular nodal blockers in these patients, there is presently no precise information available. It is unknown if there will be more harm in the future, we must also take into consideration the patient's comorbidities such as atrial fibrillation, coronary artery disease and other tachyarrhythmias. A joint discussion between the patient and the doctor is necessary when considering the restart of beta-blockers or atrioventricular nodal-blocking agents, particularly in patients who require goal-directed therapy (as part of heart failure management), as was the case with our patient.
